# Impact of Hemodialysis on Early and Long-Term Outcomes After Femoral Endarterectomy for Occlusive Disease

**DOI:** 10.3390/jcm15072796

**Published:** 2026-04-07

**Authors:** Ai Kazama, Yohei Yamamoto, Tsuyoshi Ichinose, Toru Kikuchi, Toshifumi Kudo, Tomoyuki Fujita

**Affiliations:** 1 Division of Vascular Surgery, Department of Cardiovascular Surgery, Institute of Science Tokyo, 1-5-45 Yushima, Bunkyo-ku, Tokyo 113-8519, Japan; y-yamamoto.srg1@tmd.ac.jp (Y.Y.); ichinose.srg2@tmd.ac.jp (T.I.); t-kudo.srg1@tmd.ac.jp (T.K.); 2 Division of Vascular Surgery, Nissan Tamagawa Hospital, 4-8-1 Seta, Setagaya-ku, Tokyo 158-0095, Japan; tkiksrg2@tmd.ac.jp; 3 Department of Cardiovascular Surgery, Institute of Science Tokyo, 1-5-45 Yushima, Bunkyo-ku, Tokyo 113-8519, Japan; tfujita.cvsg@tmd.ac.jp

**Keywords:** femoral endarterectomy, hemodialysis, peripheral artery disease, end-stage renal disease, chronic kidney disease, common femoral artery, open revascularization, prognosis

## Abstract

**Objectives:** To investigate the early- and long-term outcomes of femoral endarterectomy (FEA) in patients undergoing hemodialysis (HD) compared with patients who were not. **Methods:** This single-center retrospective study evaluated 199 limbs of 163 patients (HD group, *n* = 50; non-HD group, *n* = 149) who underwent elective FEA between 2013 and 2023. Clinical outcomes were compared between the groups. The primary outcomes included early postoperative morbidity and mortality, primary patency (PP), freedom from major amputation (FFMA), overall survival (OS), and amputation-free survival (AFS). **Results:** Early morbidity rates were similar between the groups. However, the mortality rate was significantly higher in the HD group than in the non-HD group. There was no significant difference in the 5-year PP rate between the two groups. Furthermore, FFMA, OS, and AFS were significantly lower in the HD group than in the non-HD group. **Conclusions:** FEA provides acceptable patency outcomes in patients undergoing HD. However, these patients have a higher early postoperative mortality rate and experience significantly poorer limb salvage and OS than non-HD patients. These findings highlight the necessity of careful perioperative management and vigilant long-term follow-up in this high-risk patient population.

## 1. Introduction

Patients with chronic kidney disease, particularly those undergoing hemodialysis (HD), constitute a rapidly expanding population at high risk for vascular surgery [1]. The prevalence of systemic atherosclerotic disease, especially peripheral artery disease (PAD), is markedly higher in patients undergoing HD than in the general population [2]. The pathophysiology of PAD in patients undergoing HD is mainly attributable to accelerated atherosclerosis, severe medial arterial calcification, chronic inflammation, and endothelial dysfunction associated with uremia [3]. Consequently, these patients frequently present with advanced PAD, including chronic limb-threatening ischemia (CLTI), and often require lower extremity revascularization. The common femoral artery (CFA) occupies a unique anatomical and functional position in lower extremity revascularization. As a critical conduit supplying both the superficial femoral artery (SFA) and the profunda femoris artery (PFA), durable reconstruction of the CFA is essential for maintaining adequate inflow and preserving collateral circulation. Femoral endarterectomy (FEA) remains a foundational surgical procedure for treating CFA occlusive disease, offering durable long-term patency [4,5,6] and reliable inflow for hybrid procedures [5,7]. Contrary to endovascular treatment, FEA enables direct removal of heavily calcified plaque and reconstruction of the CFA bifurcation, without leaving a permanent endovascular device. This is particularly advantageous in patients with extensive arterial calcification, a distinctive feature of HD-associated vascular disease [3]. Consequently, despite widespread adoption of endovascular-first strategies, FEA remains essential for PAD management. Contemporary comparative data have also suggested superior short-term patency of FEA compared with endovascular therapy for CFA lesions, as demonstrated in the CAULIFLOWER study [8].

Previous research on FEA has mainly focused on its patency [6], perioperative complications [9], surgical techniques [10], and comparison with the endovascular approach [8,11]. However, no studies have investigated the impact of dialysis as a risk factor. Specifically, it is unclear whether the excellent technical durability of FEA translates into acceptable long-term limb-related outcomes and survival in patients undergoing HD. Therefore, this study aimed to investigate early postoperative and long-term outcomes of FEA in patients undergoing HD compared with patients who were not. We assessed perioperative morbidity and mortality, primary patency (PP), freedom from major amputation (FFMA), overall survival (OS), and amputation-free survival (AFS) to better define the role of FEA in this high-risk population, thereby informing optimal perioperative management and long-term surveillance strategies.

## 2. Materials and Methods

### 2.1. Study Design and Patient Population

This retrospective observational study was conducted at a single tertiary university hospital in Japan. The study population was identified from a prospectively maintained institutional vascular surgery database and included consecutive patients who underwent elective FEA for atherosclerotic occlusive CFA disease (January 2013–December 2023). Patients were eligible for study inclusion if they underwent FEA for symptomatic PAD, including lifestyle-limiting claudication (Rutherford classification 3) or CLTI (defined as Rutherford classification ≥ 4, including ischemic rest pain, nonhealing wounds, or gangrene attributed to arterial occlusive disease). Throughout the study period, our treatment strategy remained consistent, with FEA as the first-line treatment for CFA lesions rather than endovascular treatment, and operative indications were applied consistently regardless of HD status. Limb, rather than patients, were used as the unit of analysis for limb-related outcomes, and bilateral limbs were analyzed as independent observations. The study cohort was divided into two groups based on whether or not the patient was undergoing HD at the time of surgery: the HD group and the non-HD group. Cases with missing data were excluded from the respective analysis.

### 2.2. Data Collection and Definitions

Data on baseline demographic characteristics and comorbidities, as well as surgical details, were obtained from electronic medical records. HD was defined as maintenance renal replacement therapy performed at least three times per week before surgery. Early postoperative complications were defined as those occurring within 30 days after surgery, and were graded according to the Clavien–Dindo classification system. Wound complications were defined as infection, delayed wound healing, or hematoma formation at the surgical site requiring additional treatment. Major complications were defined as Clavien–Dindo grade III or higher. PP was defined as uninterrupted patency of the treated arterial segment without restenosis or occlusion requiring reintervention. Major amputation was defined as any amputation performed above the ankle joint of the treated limb. FFMA was defined as the absence of above-ankle amputation of the treated limb. OS was defined as the time from surgery to death from any cause. AFS was defined as the time from surgery to either above-ankle amputation of the treated limb or death from any cause, whichever occurred first.

### 2.3. Surgical Procedure and Perioperative Management

FEA was performed under general anesthesia through a longitudinal or oblique groin incision at the discretion of the attending surgeon. After exposure of the CFA and its bifurcation, a longitudinal arteriotomy was performed, and endarterectomy was carried out to remove atherosclerotic plaque extending to the origins of the SFA and PFA. Additional endarterectomy of branch vessels was performed when necessary to achieve adequate inflow and outflow. Arterial reconstruction was completed using patch angioplasty with autologous vein, bovine pericardial, or prosthetic patch materials, selected based on vessel diameter, availability of autologous conduit, and surgeon preference. Concomitant revascularization procedures, including bypass surgery or endovascular interventions for proximal inflow or distal outflow lesions, were performed as part of a hybrid strategy when clinically indicated. Perioperative antithrombotic therapy consisted primarily of antiplatelet agents unless contraindicated. In patients undergoing HD, perioperative dialysis scheduling and fluid management were coordinated with nephrologists to optimize volume status and minimize perioperative hemodynamic instability. The HD patients underwent HD on the day before surgery and on postoperative day 1. Heparin was used as the anticoagulant during HD. Patients undergoing peritoneal dialysis are not included. All postoperative wound care and postoperative management followed standardized institutional protocols.

### 2.4. Follow-Up and Outcome Measures

The overall mean follow-up period was 45.2 ± 37.4 months, with a mean follow-up of 47.6 ± 40.0 months in the non-HD group and 38.0 ± 27.6 months in the HD group. Patients were followed up postoperatively with regular outpatient visits at 3-month intervals during the first postoperative year and 6-month intervals thereafter. Each visit included clinical examination, ankle-brachial pressure index (ABI) measurement, and duplex ultrasonography at the surgical site. Additional imaging evaluation using Computed tomography was performed in cases with worsening clinical symptoms or stenosis of over 50% on duplex ultrasound. Reintervention was considered in patients with >70% restenosis or symptomatic >50% restenosis.

### 2.5. Statistical Analysis

All statistical analyses were performed using EZR v1.61 (Saitama Medical Centre, Jichi Medical University, Saitama, Japan). Continuous variables were presented as mean ± SD or median [interquartile range], as appropriate, and were compared using Student *t*-test or Mann–Whitney U test. Categorical variables were expressed as counts and percentages and compared using chi-square or Fisher’s exact tests. Time-dependent outcomes, including PP, FFMA, OS, and AFS, were estimated using the Kaplan–Meier method and compared between groups using the log-rank test. Statistical significance was defined as a two-sided *p*-value < 0.05.

### 2.6. Ethical Considerations

This study was approved by the Institutional Review Board of the Institute of Science Tokyo (approval No. M2023-307, 21 March 2024). Written informed consent was waived due to the retrospective nature of the study.

## 3. Results

### 3.1. Patient Characteristics

The final study cohort comprised 163 patients (199 limbs) who underwent elective FEA during the study period. The overall mean age was 72.9 ± 8.6 years, and 70.9% of the patients were male. Common commodities included hypertension (81.4%), dyslipidemia (67.3%), and diabetes mellitus (55.8%). Fifty limbs (25.1%) were treated in patients undergoing HD due to end-stage renal disease. Regarding clinical presentation, 30.2% of limbs were classified as CLTI (Rutherford category ≥ 4) (Table 1).

Stratification by HD status revealed that patients in the HD group were significantly younger than those in the non-HD group (67.71 ± 8.14 vs. 74.71 ± 7.99 years; *p* < 0.001). The HD group showed a significantly higher prevalence of coronary artery disease (66.0% vs. 31.5%; *p* < 0.001), nonambulatory status (14.0% vs. 2.0%; *p* = 0.003), and CLTI (50.0% vs. 23.5%; *p* < 0.001) than the non-HD group. Furthermore, the number of patent tibial or peroneal arteries was significantly lower in the HD group (*p* = 0.008). Other comorbidities, including hypertension, diabetes mellitus, dyslipidemia, and pulmonary disease, as well as smoking history and preoperative ABI, were comparable between the two groups (Table 2). The HD group was significantly younger than the non-HD group. The operative indications and surgical approaches were consistent regardless of HD status, and we consider it unlikely that patient selection bias led to this difference in age between the HD vs. non-HD group.

### 3.2. Surgical Details

Table 3 summarizes the surgical details. The distribution of hybrid procedures and isolated FEA was not significantly different between the two groups. The operation time was significantly longer in the HD group than in the non-HD group (325.6 ± 135.5 vs. 269.4 ± 95.8 min; *p* = 0.002). In contrast, intraoperative blood loss did not significantly differ between the groups.

### 3.3. Early Postoperative Outcomes

Early postoperative morbidity rates were comparable between the non-HD and HD groups (10.1% vs. 14.0%; *p* = 0.442). However, early postoperative mortality was significantly higher in the HD group than in the non-HD group (6.0% vs. 0.0%; *p* = 0.015). There were no significant differences in wound-related complications within 30 days between the non-HD and HD groups (2.0% vs. 3.4%; *p* = 1.000). Postoperative ABI values were also comparable between the groups, with no statistically significant differences (Table 4).

### 3.4. Long-Term Postoperative Outcomes

**Figure 1:**
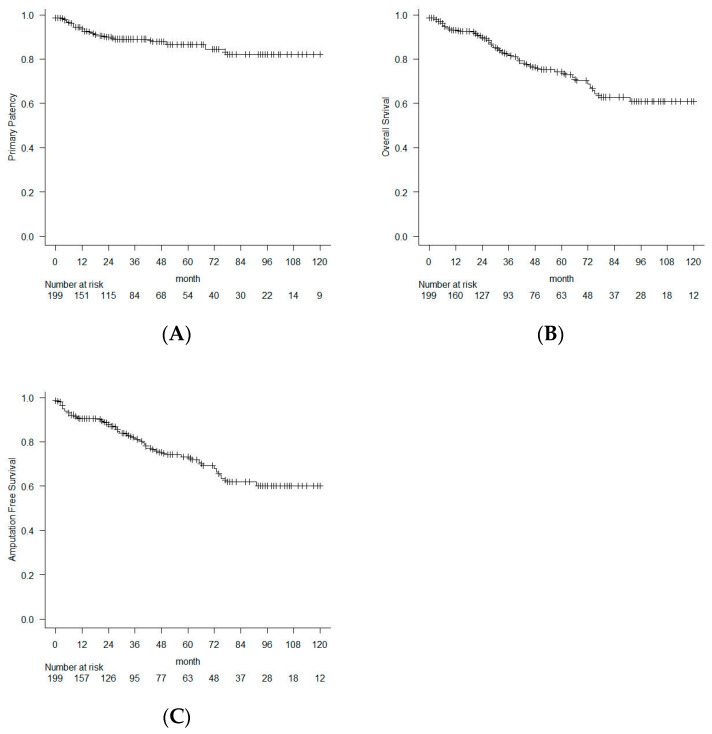
Long-term outcomes following femoral endarterectomy in the entire study population. The Kaplan–Meier curves show the (**A**) primary patency rate, (**B**) overall survival rate, and (**C**) amputation-free survival rate.

At 1, 3, and 5 years, the overall PP rate was 92.6%, 89.0%, and 86.4%; the overall OS rate was 92.7%, 82.4%, and 74.5%; and the overall AFS rate was 90.5%, 81.6%, and 73.1%, respectively (Figure 1). Throughout the entire follow-up period, the most common cause of death was heart disease (including ischemic heart disease and heart failure), followed by stroke, pneumonia, and malignant tumors.

**Figure 2:**
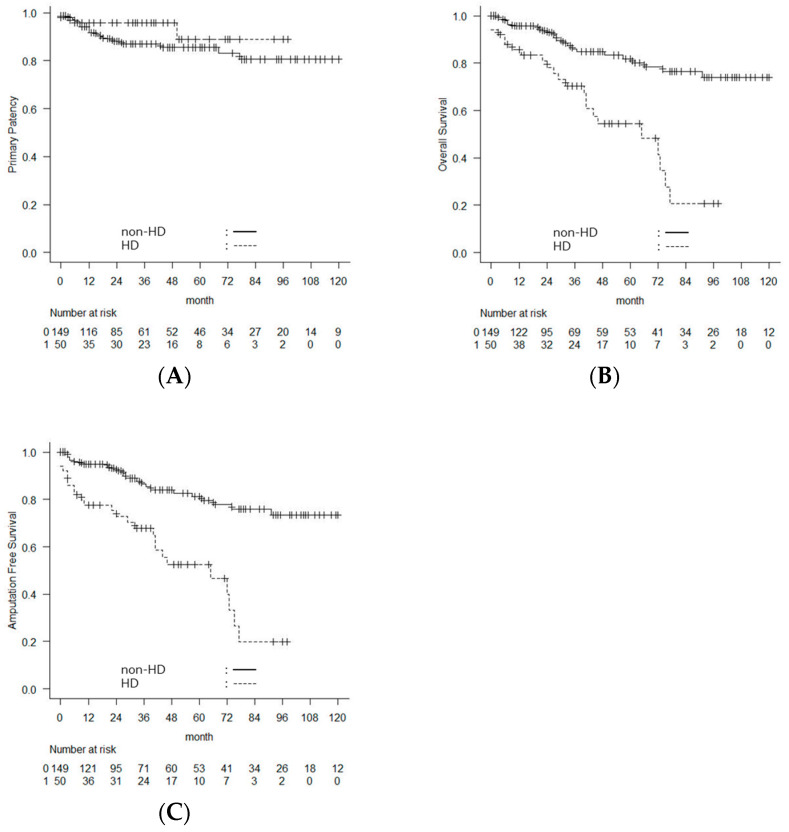
Comparison of long-term outcomes between the hemodialysis (HD) and non-HD groups. Kaplan–Meier curves comparing (**A**) primary patency, (**B**) overall survival, and (**C**) amputation free survival between patients in the HD group and those in the non-HD group after femoral endarterectomy.

Stratification of outcomes by HD status did not reveal a significant difference in PP between the two groups. The 5-year PP rate was 85.5% in the HD group and 88.9% in the non-HD group (log-rank *p* = 0.298) (Figure 2A). In contrast, OS was significantly lower in the HD group compared with the non-HD group, with a 5-year OS rate of 53.8% versus 82.4% (log-rank *p* < 0.001) (Figure 2B). Similarly, AFS was significantly lower in the HD group compared with the non-HD group, with a 5-year AFS rate of 52.4% versus 81.2% (log-rank *p* < 0.001) (Figure 2C).

**Figure 3:**
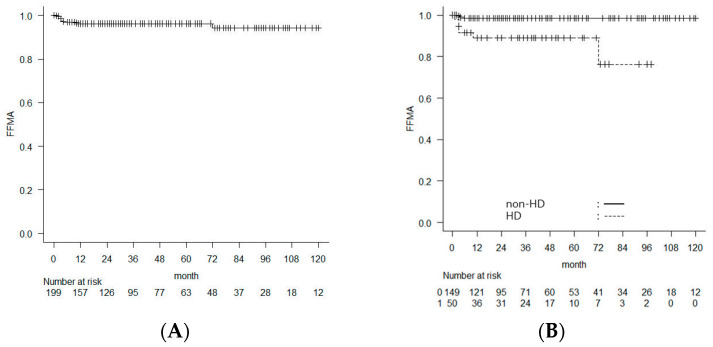
The Kaplan–Meier curves show the (**A**) freedom from major amputation (FFMA) in the entire study population and (**B**) comparison of FFMA between the hemodialysis (HD) patients and non-HD patients.

FFMA was also significantly lower in the HD group than the non-HD group, with a 5-year FFMA rate of 89.0% versus 98.5% (log-rank *p* = 0.004). Kaplan–Meier analysis revealed a significantly higher incidence of major amputation in the HD group compared with the non-HD group (Figure 3).

The results of uni- and multi-variate analysis for FFMA and OS are shown in Table 5 and Table 6. These results suggest that HD is an independent risk factor for FFMA and OS.

## 4. Discussion

The present study evaluated the impact of HD on early and long-term outcomes following FEA for atherosclerotic occlusive disease of the CFA. The patients in the HD group were significantly younger than in the non-HD group, suggesting accelerated atherosclerosis in the patients undergoing HD. Several clinically important findings emerged from this analysis. First, FEA achieved excellent long-term PP, and patency rates were comparable between patients undergoing HD and those not receiving HD. Second, although early postoperative morbidity rates were similar between the two groups, early postoperative mortality was significantly higher in the HD group. Third, long-term limb-related outcomes, including FFMA and AFS, as well as OS, were markedly worse in patients undergoing HD. Collectively, these findings highlight a critical dissociation between technical durability and long-term clinical outcomes in this high-risk population.

One of the most notable findings of this study was the preservation of excellent PP following FEA in patients undergoing HD. Despite the presence of advanced systemic atherosclerosis and severe arterial calcification, the long-term patency of the reconstructed CFA did not differ significantly between the HD and non-HD groups. This observation is consistent with previous studies demonstrating that FEA provides durable long-term patency, with reported 5-year primary patency rates exceeding 80% [4,5,6,7]. The ability of FEA to achieve durable patency in patients undergoing HD may be explained by several procedural and anatomical factors. First, direct surgical removal of atherosclerotic plaque allows for complete elimination of heavily calcified lesions that are frequently resistant to endovascular dilation. Second, the absence of a permanent endovascular device avoids issues related to stent fracture, restenosis, and impaired arterial compliance, which are particularly problematic in calcified vessels. This is particularly relevant given that patients undergoing HD commonly show a high rate of severe medial arterial calcification [3], a condition in which endovascular interventions may be technically challenging or suboptimal [12].

These findings underscore the continued relevance of FEA in the endovascular era, especially in patients with severe calcification or complex bifurcation disease. Importantly, our data suggest that HD itself does not compromise the technical success or long-term patency of FEA, reinforcing its role as a durable revascularization strategy for CFA disease even in patients undergoing HD.

In contrast to patency outcomes, early postoperative mortality was significantly higher in the HD group, despite comparable overall morbidity rates. This discrepancy suggests that patients undergoing HD are particularly vulnerable to severe postoperative complications that may rapidly become fatal. In the present cohort, causes of early postoperative death included postoperative bleeding, acute heart failure, and stroke, all of which reflect the fragile cardiovascular and hemostatic status of patients undergoing HD. Our findings emphasize that meticulous perioperative management, including careful fluid control, optimization of cardiac function, and vigilant monitoring for bleeding and thromboembolic events, is essential when performing FEA in this population.

In addition, survival and limb-related outcomes were significantly worse in the HD group than in the non-HD group. Patients in the HD group exhibited markedly reduced OS and AFS, as well as a higher incidence of major amputation. These outcomes align with extensive prior literature reporting poor outcomes after lower extremity revascularization in patients with end-stage renal disease [13]. Several mechanisms may account for these inferior outcomes. Orimoto et al. reported that poor nutritional condition due to low energy intake and chronic inflammation contributes to excess mortality and limb loss in this population [14]. Moreover, the significantly higher proportion of CLTI and nonambulatory status in the HD group reflects a more advanced disease state at presentation, which may further exacerbate adverse outcomes.

The findings of this study have important implications for clinical decision-making in patients undergoing HD who require revascularization for CFA disease. From a clinical perspective, our findings support FEA as a durable and effective revascularization strategy for CFA disease in patients undergoing HD with regard to vessel patency. However, the significantly reduced long-term survival and limb salvage rates underscore the importance of individualized decision-making incorporating functional status, life expectancy, and patient preferences. In selected high-risk patients with limited life expectancy or severe frailty, less invasive endovascular strategies may be considered; however, further studies are required to determine the optimal treatment approach in this population.

Given the heterogeneity of outcomes observed in patients undergoing HD, future research should focus on identifying reliable prognostic markers to improve risk stratification and patient selection. Biomarkers of systemic inflammation, nutritional status, and frailty may help predict long-term outcomes following FEA. In this context, our group recently reported the neutrophil-to-lymphocyte ratio as a significant prognostic marker of clinical outcomes in patients undergoing FEA [15]. Similarly, further studies are warranted to identify prognostic factors for predicting clinical outcomes in patients undergoing HD. Furthermore, multicenter prospective studies with larger cohorts of patients undergoing HD are warranted to validate the findings of the present study and to explore the comparative effectiveness of different revascularization strategies in this population. Such efforts may ultimately contribute to the development of evidence-based guidelines tailored to the unique challenges posed by patients with end-stage renal disease.

This study has several limitations. First, its retrospective, single-center design introduces the potential for selection bias and unmeasured confounders. This research design limits its applicability to other institutions. Second, the relatively small sample size of the HD group may limit the statistical power for subgroup analyses. Third, we did not analyze cause-specific mortality, precluding a detailed assessment of the underlying mechanisms of late deaths. Despite these limitations, this study offers meaningful insights into the role of FEA in patients undergoing HD. The strengths of the study include a well-characterized cohort, detailed surgical data, and comprehensive long-term follow-up.

## 5. Conclusions

FEA provides excellent and durable long-term PP in patients undergoing HD, comparable to outcomes observed in patients without HD. These findings confirm that the technical effectiveness of FEA is preserved even in the presence of advanced systemic atherosclerosis and severe arterial calcification associated with end-stage renal disease. However, patients undergoing HD experience significantly higher early postoperative mortality and markedly inferior long-term survival and limb-related outcomes, including AFS and FFMA. These results indicate that, while FEA is a reliable revascularization strategy from a technical standpoint, overall prognosis in this population is predominantly determined by systemic disease burden rather than local vascular durability alone. Careful patient selection, meticulous perioperative management, and realistic goal setting that incorporates functional status, life expectancy, and patient preferences are essential when considering FEA in patients undergoing HD. Further prospective, multicenter studies are warranted to refine risk stratification and optimize treatment strategies for this vulnerable population.

## Figures and Tables

**Table 1:** Demographics and preoperative comorbidities of the study population.

	All Subjects (*n* = 199 Limbs)
Age, years	72.9 ± 8.6
Male	141 (70.9)
Hypertension	162 (81.4)
Dyslipidemia	134 (67.3)
Diabetes mellitus	111 (55.8)
Coronary artery disease	80 (40.2)
Cerebrovascular disease	33 (16.6)
Pulmonary disease	38 (19.1)
End-stage renal disease	50 (25.1)
Ever smoked	159 (79.9)
Rutherford classification	
3	137 (68.8)
4	28 (14.1)
5	28 (14.1)
6	4 (2.0)

Data are expressed as mean ± standard deviation or number (percentage).

**Table 2:** Comparison between the non-HD and HD groups.

	Non-HD Group (*n* = 149 Limbs)	HD Group (*n* = 50 Limbs)	*p* Value
Age, years	74.71 ± 7.99	67.71 ± 8.14	<0.001
Male	102 (68.5)	39 (78.0)	0.269
Hypertension	119 (79.9)	43 (86.0)	0.450
Dyslipidemia	105 (70.5)	29 (58.0)	0.146
Diabetes mellitus	80 (53.7)	31 (62.0)	0.390
Coronary artery disease	47 (31.5)	33 (66.0)	<0.001
Cerebrovascular disease	20 (13.4)	13 (26.0)	0.064
Pulmonary disease	29 (19.5)	9 (18.0)	1
Ever smoked	120 (80.5)	39 (78.0)	0.854
Non-ambulatory status	3 (2.0)	7 (14.0)	0.003
Preoperative ABI	0.52 ± 0.25	0.52 ± 0.28	0.884
Number of patent tibial or peroneal arteries			
0	10 (6.7)	13 (26.0)	
1	35 (23.5)	11 (22.0)	
2	46 (30.9)	15 (30.0)	
3	46 (30.9)	10 (20.0)	0.008
CLTI (≥Rutherford 4)	35 (23.5)	25 (50.0)	<0.001

Data are expressed as mean ± standard deviation or number (percentage). HD: hemodialysis, ABI: ankle brachial index, CLTI: chronic limb-threatening ischemia.

**Table 3:** The operative details.

	All Subjects (199 Limbs)	Non-HD Group (149 Limbs)	HD Group (50 Limbs)	*p* Values
Isolated FEA	50 (25.1)	39 (25.5)	11 (22.0)	0.758
Use of patch	167 (83.9)	125 (83.9)	42 (84.0)	1.000
Inflow revascularization	98 (49.2)	78 (52.3)	20 (40.0)	0.206
Outflow revascularization	88 (44.2)	63 (42.3)	25 (50.0)	0.432
Surgical operating time	283.51 ± 109.5	269.4 ± 95.8	325.6 ± 135.5	0.002
Amount of bleeding	455.5 ± 592.0	437 ± 657.5	507 ± 325.9	0.471

Data are expressed as the number (percentage) or mean ± standard deviation. HD: hemodialysis, FEA: femoral endarterectomy.

**Table 4:** The early postoperative outcomes.

	All Subjects (199 Limbs)	Non-HD Group (149 Limbs)	HD Group (50 Limbs)	*p* Values
Postoperative hospital stay, days	10 (9–13)	9 (8–13)	10.5 (7–21.25)	0.092
Postoperative ABI	0.88 ± 0.21	0.89 ± 0.20	0.82 ± 0.26	0.136
Major amputation	8 (4.0)	2 (1.3)	6 (12.0)	0.004
Postoperative complications within 30 days	22 (11.1)	15 (10.1)	7 (14.0)	0.442
Clavien-Dindo	12 (6.0)	6 (4.0)	6 (12.0)	0.078
Classification ≥ III				
Wound complication	6 (3.0)	5 (3.4)	1 (2.0)	1
Bleeding	8 (4.0)	4 (2.6)	3 (6.0)	0.371
Heart failure	1 (0.5)	0 (0.0)	1 (2.0)	0.251
Stroke	3 (1.5)	1 (0.7)	2 (4.0)	0.156
Postoperative mortality within 30 days	3 (1.5)	0 (0.0)	3 (6.0)	0.015

Data are expressed as the number (percentage) or median value (interquartile range). ABI: ankle brachial index.

**Table 5:** Uni- and multi-variate analysis for FFMA.

FFMA		Univariate			Multivariate	
	HR	95% CI	*p* Value	HR	95% CI	*p* Value
Female	3.904	0.932–16.350	0.062	5.386	1.270–22.830	0.022
Hypertension	1.910	0.234–15.750	0.544			
Dyslipidemia	3.498	0.430–28.440	0.242			
Diabetes mellites	5.860	0.707–48.500	0.101	4.650	0.568–38.080	0.152
Coronary artery disease	2.580	0.598–11.100	0.204			
Cerebrovascular disease	1.600	0.324–7.945	0.563			
Hemodialysis	10.00	1.950–51.400	0.006	12.840	2.551–64.590	0.002
Non-ambulatory status	7.620	1.330–43.900	0.002	2.495	0.437–14.260	0.304
CLTI (≥Rutherford 4)	2.410	0.582–9.980	0.225			

FFMA: free from major amputation, HR: hazard ratio, CI: confidence interval, CLTI: chronic limb-threatening ischemia.

**Table 6:** Uni- and multi-variate analysis for overall survival.

Overall Survival		Univariate			Multivariate	
	HR	95% CI	*p* Value	HR	95% CI	*p* Value
Female	1.092	0.580–2.056	0.784			
Hypertension	2.624	1.031–6.674	0.043	2.539	0.983–6.559	0.054
Dyslipidemia	0.859	0.470–1.570	0.620			
Diabetes mellites	1.590	0.800–3.170	0.185	0.609	0.300–1.236	0.170
Coronary artery disease	1.250	0.641–2.450	0.510			
Cerebrovascular disease	2.480	1.346–4.568	0.004	1.432	0.718–2.856	0.308
Hemodialysis	4.920	2.400–10.100	<0.001	2.731	1.466–5.086	0.002
Non-ambulatory status	1.500	0.372–6.050	0.569			
CLTI (≥Rutherford 4)	3.750	1.870–7.500	<0.001	3.261	1.751–6.074	<0.001

HR: hazard ratio, CI: confidence interval, CLTI: chronic limb-threatening ischemia.

## Data Availability

The data that support the findings of this study are available from the corresponding author.

## Abbreviations

The following abbreviations are used in this manuscript:

ABI	Ankle brachial index
AFS	Amputation free survival
CFA	Common femoral artery
CLTI	Chronic limb-threatening ischemia
FEA	Femoral endarterectomy
FFMA	Free from major amputation
HD	Hemodialysis
OS	Overall survival
PAD	Peripheral artery disease
PP	Primary patency
PFA	Profunda artery
SFA	Superior femoral artery
